# The effectiveness and cost-effectiveness of Acceptance and Commitment Therapy as a transdiagnostic intervention for transitional-age youth: study protocol of a randomized controlled trial

**DOI:** 10.1186/s12888-023-04535-z

**Published:** 2023-01-19

**Authors:** Janna Keulen, Denise Matthijssen, Jacquelijne Schraven, Maja Deković, Denise Bodden

**Affiliations:** 1 Department of Clinical Child & Family Studies, Utrecht University, Utrecht, The Netherlands; 2 Altrecht Child and Youth Psychiatry, Utrecht, The Netherlands

**Keywords:** Acceptance and commitment therapy, Transitional-age youth, Randomized controlled trial, Transdiagnostic intervention, Effectiveness, Cost-effectiveness

## Abstract

**Background:**

Although the prevalence of psychological problems in transitional-age youth (i.e., youth aged 15 to 25; TAY) is high, TAY are much less likely to receive age-appropriate treatments for their psychological problems compared to younger adolescents or older adults. Hence, effective interventions for TAY seem warranted. ACT your way is a transdiagnostic treatment, specifically developed for TAY, based on the principles of Acceptance and Commitment Therapy (ACT). ACT your way is not directed primarily at symptom reduction, but mainly aims to change the underlying mechanism of psychopathology, namely increasing TAY’s psychological flexibility. Meta-analyses show that ACT is an effective treatment for adults with diverse types of psychopathology. Less is known about the effectiveness of ACT for TAY. Therefore, the goal of this study is to examine the effectiveness and cost-effectiveness of ACT your way. In addition, we will investigate for whom and under what circumstances (i.e., moderators) and how (i.e., mediators) the intervention is (most) effective.

**Method:**

The study is designed as a multi-centre, randomized controlled trial. In total, 140 TAY diagnosed with any psychological disorder will be randomly assigned to either the ACT your way or treatment as usual (TAU) condition. In total, six assessments will be conducted: at baseline, after 3, 6 and 9 sessions, at post-intervention and at 6-month follow-up, using multiple informants (TAY, parents/caregivers, therapists). Assessments will include diagnostic interviews and questionnaires. The primary outcomes are psychological flexibility and number of DSM-5 diagnoses; the secondary outcomes are the presence of the primary DSM-5 diagnosis, psychopathology, personality problems, global, individual and societal functioning, quality of life, stress, treatment satisfaction, treatment drop-out and therapeutic alliance. We will also assess costs and various moderators (i.e., demographic characteristics, type and severity of problems, psychopathology of parents/caregivers, treatment expectancy and previous treatments) and mediators (i.e., psychological flexibility, emotion regulation, self-compassion, autonomy, perfectionism, self-esteem and group cohesion).

**Discussion:**

To our knowledge, this is the first study investigating the (cost-)effectiveness of ACT compared to TAU in clinically referred TAY with various types of psychopathology, using a rigorous design.

**Trial registration:**

The research project is registered in the Dutch Trial Register (Trial NL9642).

The developmental period of middle adolescence to early adulthood is a crucial period characterized by significant variability and change. During this period, youths make many important life choices and develop various habits that could affect their later (mental) health [1]. Moreover, in this life stage, youths face numerous developmental and psychosocial challenges, such as finishing their education, choosing a career, building new (romantic) relationships and becoming self-reliant. Also, psychological restructuring takes place that is often associated with identity crisis, risk behaviour and emotional instability [2]. Due to these significant and often stressful changes, transitional-age youth (i.e., youth aged 15 to 25; TAY) are at great risk of developing new psychological problems or persisting to experience psychological problems they developed during childhood [2, 3]. To illustrate, in 2021, eighteen percent of the Dutch adolescents and young adults indicated to be mentally unhealthy (e.g., stressed, anxious or depressed) [4]. Moreover, results from the global burden of disease study show that the burden of psychological disorders peaks between middle adolescence and young adulthood [5]. These elevated prevalence rates of psychological disorders in TAY are problematic, as many psychological disorders persist into adulthood, cause comorbid disorders [6–8] and are strongly related to other health and developmental concerns in youths (e.g., lower educational and work achievements, troubles with law, social isolation, suicide, substance abuse and violence) [6, 9]. Hence, the transitional-age period is a particularly important period for intervening effectively.

Although the prevalence of psychological problems in TAY is high, TAY are much less likely to receive treatment for their psychological problems compared to younger adolescents or older adults [10, 11]. Moreover, when TAY receive treatment for their psychological problems, they are significantly more likely to drop-out compared to older adults [12]. Also, in many countries, the collaboration between youth (i.e., below the age of 18) and adult (i.e., 18 years or older) mental health care facilities is not optimal, resulting in no or poor transitions from youth- to adult care after adolescents turn 18. These poorly executed transitions threaten the continuity of psychological care for TAY in this already challenging and critical developmental period [13, 14]. Additionally, when TAY are referred for treatment, they often receive evidence-based interventions developed for either youth (i.e., below the age of 18) or adults (i.e., 18 years or older) [15, 16]. These evidence-based interventions do not meet TAY’s needs and are often not developmentally sensitive. Therefore, effective interventions specifically developed for TAY seem warranted.

There are several important aspects that could be considered when developing a suitable treatment for TAY. First, in a qualitative study, TAY indicated that an important barrier for active participation in mental health care was having a therapist who acts as an authority figure, has little interest in the TAY’s perspective and does not stimulate the TAY to participate in the decision making [17]. Hence, when developing interventions for TAY, it is important to consider the TAY’s perspective and stimulate TAY to participate in framing the therapy. Second, during the transitional-age period, TAY need to form an identity and become self-reliant autonomous figures [2]. Therefore, a strong focus on identity and autonomy development within interventions for TAY seem warranted. Third, as the transitional-age period is a period of significant variability and change, TAY’s psychological problems are often complex and changeable. For instance, during the course of treatment new stressors, associated with various developmental challenges (e.g., finishing education, moving out of their parental home or ending a romantic relationship), often arise. These stressors could potentially cause the development of new or comorbid problems and alter TAY’s therapy needs [18]. Most current evidence-based interventions only focus on treating symptoms of one specific disorder, making these interventions less suitable for TAY with changing symptom profiles and comorbid problems. For these TAY, interventions targeting transdiagnostic mechanisms that underly the symptoms of multiple disorders might be more suitable. Such transdiagnostic interventions have a better fit with the complex and changeable psychological problems most TAY experience. Moreover, transdiagnostic interventions have potential benefits over traditional interventions as various psychological symptoms can be treated within one intervention, possibly contributing to the efficiency and (cost-)effectiveness of care [19]. Fourth, most current evidence-based interventions focus on the reduction of symptoms, making these interventions less suitable for TAY with recurrent of chronic psychological problems. For these TAY, interventions that focus on increasing quality of life or learning skills that help to build resilience might be more applicable.

ACT your way is a transdiagnostic intervention specially developed for TAY. Within ACT your way, therapists and TAY contribute equally to framing therapy goals and actions, possibly stimulating TAY to actively participate within the intervention. Furthermore, due to its strong focus on identity and autonomy development, the intervention fits well with the developmental needs of this age group. Moreover, the intervention is not aimed primarily at symptom reduction, but mainly at increasing quality of life and changing the underlying mechanisms of psychopathology. This makes the intervention also suitable for TAY with changing symptom profiles, comorbidity, and chronic or recurrent psychological problems.

ACT your way is based on the principles of Acceptance and Commitment Therapy (ACT). The main purpose of ACT is to increase psychological flexibility, a transdiagnostic mechanism which can be defined as an individual’s acceptance of negative feelings, thoughts and physical sensations, and the ability to choose an adaptive (and more effective) response [20]. More specifically, psychological flexibility contains six core processes: acceptance (i.e., learning to accept unpleasant emotions, thoughts and situations, instead of avoiding or fighting against them), defusion (i.e., changing the unwanted functions of thoughts, instead of changing their form, frequency or sensitivity), contact with the present moment (i.e., learning to focus on the here-and-now, instead of ruminating about the past or worrying about the future), self as context (i.e. developing a flexible view of the self, where content about the self can be observed and accepted), values (i.e., determining what is most important for oneself), and committed action (i.e., changing behaviour in the direction of ones values) [20]. In ACT, these six core processes are stimulated using psychoeducation, mindfulness exercises, metaphors and experiential exercises [20].

There are several meta-analyses showing that ACT is equally effective compared to well established evidence-based treatments (e.g., cognitive behavioral therapy; CBT) and superior to inactive control conditions (e.g., placebo or waitlist) and treatment as usual (TAU) in adults with depression, anxiety, pain, substance use and other types of psychopathology [21–24]. Moreover, in the United States, ACT is registered as evidence-based treatment for adults with chronic pain and depression [25]. Until now, less is known about the effectiveness of ACT for adolescents and young adults [26, 27]. Few studies investigated the effectiveness of ACT in adolescents and/or young adults with different types of problems, such as anorexia nervosa [28, 29], obsessive–compulsive anxiety [30], pain [31, 32], depressive symptoms [33, 34], autism spectrum disorder [35], social and school adaptation [36], social anxiety [37], posttraumatic stress [38], learning disabilities and anxiety [39], academic procrastination [40], trichotillomania [41], aggression [42] and antisocial behaviour [43]. These studies show promising effects. In addition, the effectiveness of the ACT your way intervention has recently been examined in a pilot study with 23 TAY with recurrent or chronic depression, who previously received CBT [44]. At posttreatment, 76.2% of the TAY did not meet the criteria for a depressive disorder anymore. Moreover, participants reported significant improvements in depressive symptoms, quality of life, perceived competence, and comorbid internalizing problems. In addition, the treatment drop-out was remarkably less (20.6%) compared to treatment drop-out in a CBT trial for the same target group of 12 to 21 year olds (ranging from 41 to 57%) [45]. Hence, results in terms of the effectiveness of ACT your way specifically, also seem promising. However, many of the abovementioned studies lack an adequate sample size, do not use a control group or randomisation, or are case studies. Furthermore, no research has been done in TAY with a wide range of psychological disorders, including comorbid, chronic and/or recurrent diagnoses. In addition, the cost-effectiveness of ACT in TAY has never been studied before.

Therefore, the first aim of this study is to evaluate the effectiveness of ACT your way in TAY with a wide range of psychological disorders. In this study, ACT your way will be compared to TAU. We expect that, compared to TAU, ACT your way shows more improvements in our primary (i.e., psychological flexibility and the number of DSM-5 diagnoses) and secondary outcomes (i.e., the presence of the primary DSM-5 diagnosis, psychopathology, personality problems, global functioning, individual and societal functioning, quality of life, stress) at the short term (immediately after the intervention), but also at the long term (after six months). Furthermore, we expect more treatment satisfaction, less treatment drop-out and better treatment alliance (i.e., also secondary outcomes) of TAY since ACT your way is more attuned to their needs. The second aim is to examine the cost-effectiveness of ACT your way. We expect that ACT your way is more cost-effective than TAU, because, compared to carrying out multiple diagnosis-specific interventions, ACT your way can treat various problems at once [18].

The third aim is to investigate for whom and under what circumstances ACT you way works best by testing possible moderators (i.e., demographic characteristics [age, gender, education level and ethnicity of TAY], type and severity of problems, psychopathology of parents/caregivers, treatment expectancy and previous treatments). Moderators are baseline characteristics that interact with the type of treatment (i.e., ACT your way or TAU) to affect outcome. As most prior studies investigating the effects of ACT do not compare ACT to active treatments, the knowledge on treatment moderators is scarce. Based on existing findings, we expect that TAY with severer problems (i.e., higher baseline levels of psychopathology and/or more comorbid problems) will benefit more from ACT your way than from TAU [46–49]. Due to contrasting (e.g., [47, 50]) or insufficient findings in earlier research, no hypotheses are formulated regarding the moderating roles of demographic characteristics (age, gender, education level and ethnicity of TAY), type of problems, psychopathology of parents/caregivers, treatment expectancy and the number of previous treatments.

The fourth aim is to examine through which mechanisms ACT your way works by testing possible mediators (i.e., psychological flexibility, emotion regulation, self-compassion, autonomy, perfectionism, self-esteem and group-cohesion). Based on studies investigating mediators of the effectiveness of ACT, we hypothesize that ACT your way will increase TAY’s psychological flexibility [51, 52], emotion regulation [53, 54] and self-compassion [55, 56] which, in turn, will affect the primary (e.g., number of DSM-diagnoses) and secondary outcomes (e.g., quality of life). To our knowledge, there are no studies investigating if the effects of ACT are mediated by the clients’ autonomy, perfectionism, self-esteem and group cohesion. Notwithstanding, there are studies showing that ACT can decrease the clients’ perfectionism [57, 58] and increase the clients' self-esteem [40, 44, 59, 60], and that individuals with higher levels of perfectionism and lower levels self-esteem more often experience psychological problems than individuals with lower levels of perfectionism and higher levels of self-esteem [61, 62]. Hence, we also expect that ACT your way will decrease TAY’s perfectionism and will increase TAY’s self-esteem, which, in turn, will improve the primary (e.g., number of DSM-diagnoses) and secondary outcomes (e.g., quality of life). Due to insufficient findings in earlier research, no hypotheses are formulated regarding the mediating roles of autonomy and group cohesion.

## Method

The study will be reported in accordance with the CONSORT 2010 statement [63]. The research project is approved by the Medical Research Ethics Committee NedMec (NL78679.041.21) and registered in the Dutch Trial Register (Trial NL9642).

### Participants

In total, 140 TAY with any psychological disorder (e.g., anxiety disorder, obsessive–compulsive disorder (OCD), trauma, depressive disorder, persistent depressive disorder, oppositional defiant disorder (ODD), conduct disorder (CD), personality disorder, autism spectrum disorder or any combination of these) will be included in the study. Also their parents/caregivers and therapists will be asked to participate. Participants will be recruited between March 2022 and March 2024.

#### Inclusion and exclusion criteria

Inclusion criteria for the TAY are (1) having a psychological disorder, (2) being 15 to 25 years old, (3) being referred to one of the participating mental healthcare institutions. Exclusion criteria are (1) having insufficient knowledge of the Dutch language, (2) acute suicide risk, (3) currently meeting the criteria for a DSM-5 substance abuse, psychotic and/or bipolar disorder, (4) having an estimated IQ below 80, (5) unstable medication (i.e., the medication should be set before the start of the intervention and should remain stable during the intervention), (6) absence of TAY’s or parental permission (for adolescents below the age of 16).

#### Sample size calculation

We calculated the sample size for a two-level multilevel analysis, using the Optimal Design program [64]. In accordance with our planned analyses, the first level represents the TAY and the second level represents the therapists. As most of the therapists either give the ACT your way or the TAU intervention, we calculated the sample size as for a cluster RCT in which the therapists represent the clusters. Within our sample size calculation, we used a cluster size (i.e., the expected number of therapists) of 70, as we expect that in both conditions approximately 35 therapists will be carrying out either the ACT your way or TAU intervention. In addition, we used an ICC of 0.05 as this is the average ICC reported in meta-analyses of therapist effects in psychological intervention research [65]. Furthermore, the effect size is based on a meta-analysis in which 39 RCT’s were included that investigated the efficacy of ACT, including 1,821 adults with various mental disorders [21]. We based the effect-size on this meta-analysis as, to our knowledge, this is the most recent meta-analysis examining the effects of ACT transdiagnostically. When ACT was compared to TAU a medium effect was found (Hedges g = 0.64), when ACT was compared to CBT a small effect was found (Hedges g = 0.32). In this study ACT will be compared to TAU, presuming a medium effect around 0.64. However, since we want to be conservative and expect that, at least with some of the participants, CBT will be used in TAU as well, an effect size of 0.50 was included in the sample size calculation. Hence, with a cluster size of 70, an effect size of 0.50, an ICC of 0.05, an α of 0.05 and a power of 0.8, a sample size of 140 (i.e., on average two TAY per therapist, and 70 TAY per condition) is assumed.

### Study design and procedure

The study is designed as a multi-center, randomized controlled trial (RCT). Data will be collected in 14 Dutch and Belgian mental healthcare institutions. Within each institution, TAY meeting the inclusion/exclusion criteria, their parents/caregivers and their therapists will be asked to participate in the study. Parents/caregivers of adolescents younger than 16 will be directly involved in the study. TAY aged 16 and older will be asked first for permission to contact their parents/caregivers. If they agree, their parents/caregivers will be asked to participate in the study as well. Before enrolling in the study, written informed consent from TAY and parents/caregivers/legal representatives (for adolescents younger than 16) will be obtained. If parents/caregivers and therapists agree to participate, they will be asked for their own written informed consent as well.

**Fig. 1:**
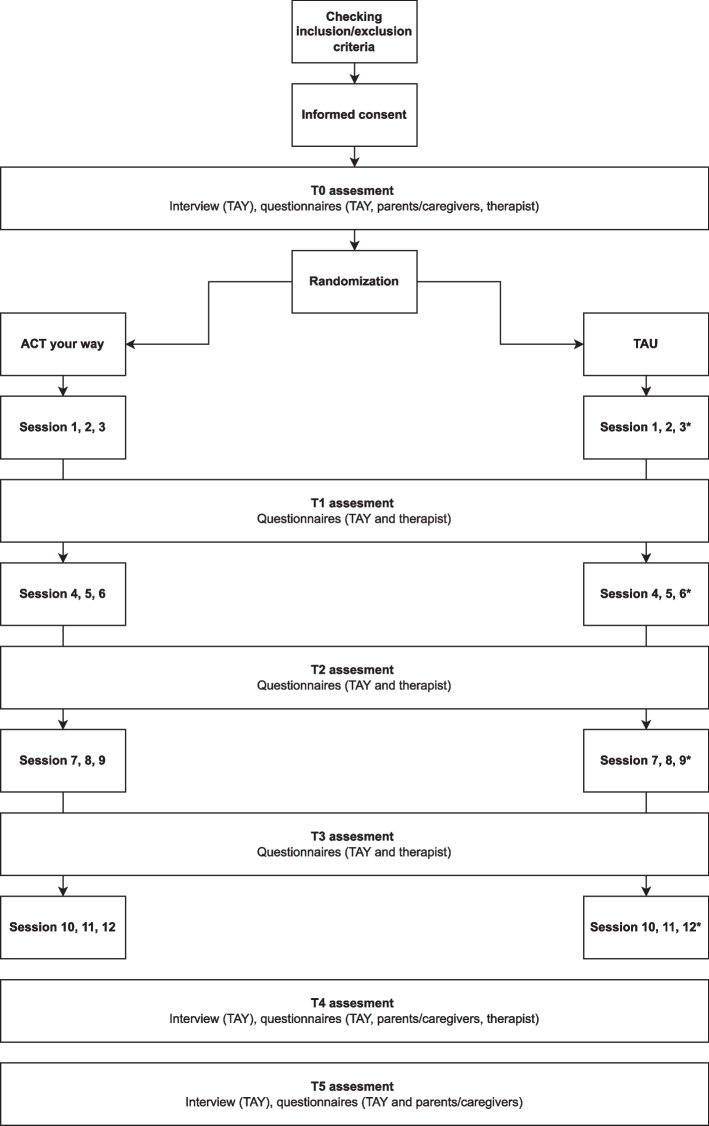
Overview of study design. *Only if TAU is carried out weekly. If TAU is carried out bi-weekly, the T1, T2, T3 and T4 take place after 2, 4, 6 and 8 sessions, respectively

In total, six multiple informant (TAY, parents/caregivers, therapist) assessments will be conducted: at baseline (T0), after 3 sessions (T1), after 6 sessions (T2), after 9 sessions (T3), at post-intervention (T4) and at 6-month follow-up (T5). Assessments will include diagnostic interviews (at T0, T4 and T5) and questionnaires (at T0 to T5). The assessments will be conducted by research assistants working at the participating institutions (e.g., master students and psychologists). Research assistants will be trained in administering the diagnostic interview and questionnaires. The TAY will receive a 30 euro gift card as a reward (5 euros per assessment). After the baseline assessment (T0), TAY will be randomly assigned to either the ACT your way or TAU condition using a computer generated block design. At each institution, five participants will be randomly allocated to the ACT your way condition and five participants will be randomly allocated to the TAU condition (blocksize = 10). See Fig. 1 for an overview of the study design.

### Study conditions

#### ACT your way

ACT your way consists of 12 weekly sessions, that can be carried out both individually as in groups. The therapists at the clinical institutions decide if they carry out ACT your way individually or in a group. There is a guide for therapists [66], a workbook for TAY [67] and a website with additional videos, mindfulness exercises and information for TAY. Each session lasts either 120 (group therapy) or 60 (individual therapy) minutes and has a fixed format in which psychoeducation, mindfulness exercises, metaphors and experiential exercises are carried out. The 12 sessions are divided into four blocks, each comprising of three sessions. The first block focusses on values (i.e., desires regarding development, identity, and autonomy). The second block focusses on the six core processes of psychological inflexibility (i.e., experiential avoidance, cognitive fusion, dominance of conceptualized past and feared future, attached to the conceptualised self, lack of values, and inaction). The third block focuses on acquiring skills based on the six core processes of psychological flexibility (i.e., acceptance, cognitive defusion, contact with the present moment, self as context, values, and committed action). With these skills, TAY can consciously choose behaviours that fit their chosen values. The fourth block focusses on continuing and strengthening TAY’s psychological flexibility and preparing the TAY for possible relapses in the future. In this study, ACT your way will be carried out in a face-to-face format by therapists working at the participating mental healthcare institutions. The therapists will be trained in using ACT your way (i.e., a two day training in the basics of ACT and a two day training in ACT your way specifically). Moreover, during the course of the study, the therapists will be supervised by an ACT expert.

#### Treatment as usual

TAU can also be carried out both individually as in groups and will consist of a broad range of different psychological interventions, such as CBT, parent counselling, emotion regulation training, running therapy, or psychodynamic therapy. Therapists of the participating mental health care institutions decide what intervention(s) are most suitable to be carried out as TAU. We expect that in most cases, similar to ACT your way, TAU will also consist of weekly sessions. However, in the few cases that this is not possible, it is also acceptable to have bi-weekly sessions within TAU. If TAU is carried out bi-weekly, the T1, T2, T3 and T4 will take place after 2, 4, 6 and 8 sessions, respectively. We choose this option as then both in the ACT your way as TAU condition, the assessments will take place approximately around the same time.

### Measures

See Table 1 for an overview of all measures used in the study.

**Table 1:** Overview of study measures

Variable	Domain	Instrument	Items	Source			Assessment					
				**Y**	**P**	**T**	**T0**	**T1**	**T2**	**T3**	**T4**	**T5**
Primary outcomes	Psychological flexibility	AFQ-Y (FV)	17	x			x		x		x	x
		AFQ-Y (SV)	8	x				x		x		
	Number of DSM-5 diagnoses	SCID-5 Junior	-	x			x				x	x
Secondary outcomes	Presence of the primary DSM-5 diagnosis	SCID-5 Junior	-	x			x				x	x
	Psychopathology	YSR	69	x			x				x	x
		CBCL	74		x		x				x	x
		BFS	12	x			x	x	x	x	x	x
	Personality problems	SIPP-SF	60	x			x		x		x	x
	Global Functioning	CGAS	1			x	x		x		x	
		ORS	4	x			x	x	x	x	x	x
	Individual and societal functioning	ISIQ	10	x			x				x	x
	Quality of life	EuroQol	6	x	x		x		x		x	x
	Stress	PSS (FV)	10	x			x		x		x	x
		PSS (SV)	5	x				x		x		
	Treatment satisfaction	SSS	4	x							x	
	Drop-out	Drop-out questionnaire	5			x					x	
	Therapeutic alliance	TASC	12	x		x		x	x	x	x	
Moderators	Demographic characteristics	-	-	x	x	x	x				x	x
	Type of problems	SCID-5 junior	-	x			x				x	x
	Severity of problems	SCID-5 junior	-	x			x				x	x
	Psychopathology of parents/caregivers	ASR	69		x		x				x	x
	Treatment expectancy	PETS	7	x			x					
	Previous treatments	VEHI	8	x			x					
Mediators	Psychological flexibility	AFQ-Y (FV)	17	x			x		x		x	x
		AFQ-Y (SV)	8	x				x		x		
	Emotion regulation	ERSQ	27	x			x		x		x	x
		ERSQ (SV)	9	x				x		x		
		FEEL-KJ	30	x			x		x		x	x
	Self-compassion	SCS-SF	12	x			x		x		x	x
	Autonomy	AAQ	18	x			x		x		x	x
	Perfectionism	FMPS	26	x			x		x		x	x
	Self-esteem	RSES	10	x			x		x		x	x
	Group cohesion	GCQ-S	12					x	x	x	x	
Other characteristics	Treatment integrity	Observation	-									
	Content of TAU	TPC	3			x		x	x	x	x	
	Life events	LES	23	x			x				x	x
	Costs	Cost diary	79	x			x				x	x

Abbreviations: *FV* Full-length version, *P* Parents/caregivers, *SV* Short version, *T* Therapist, *T0* Baseline assessment, *T1–3* mediator assessments, *T4* Post-assessment, *T5* 6-month follow-up assessment, *Y* Transitional age youths

#### Primary outcomes

*Psychological flexibility* will be measured with the Acceptance and Fusion Questionnaire for Youth (AFQ-Y) [68, 69] at T0, T2, T4 and T5. The AFQ-Y contains 17 items that are rated on a 5-point Likert scale ranging from 0 = “*not at all true*” to 4 = “*very true*”. The psychometric properties of the AFQ-Y are demonstrated both in adolescent and adult samples [68, 70]. During T1 and T3, a short version (8 items) of the AFQ-Y will be administered (i.e., the AFQ-Y-8), which also has adequate psychometric properties [68, 69].

The *number of DSM-5 diagnoses* will be measured with the Structured Clinical Interview for DSM-5 Disorders—Junior (SCID-5-Junior) [71] at T0, T4 and T5. The SCID-5-Junior is a semi-structured diagnostic interview that assesses a wide range of DSM-5 disorders in children and adolescents. Preliminary results of studies investigating the psychometric properties of the SCID-5 junior show adequate parent–child agreements (except for externalising problems and insomnia) and an adequate convergent validity (especially in clinical samples) [71]. The SCID-5-junior will be conducted by trained research assistants working at the participating institutions. All research assistants will video/audio tape two SCID-5-junior interviews. The recorded SCID-5-junior interviews will be rated by an independent researcher to calculate the inter-rater reliability.

#### Secondary outcomes

The *presence of the primary DSM-5 diagnosis* will also be measured with the SCID-5-Junior [71] at T0, T4 and T5.

*Psychopathology* will be assessed by means of two informants: TAY will fill out the Youth Self Report (YSR) [72] at T0, T4 and T5 and parents/caregivers will fill out the Child Behavior Checklist about their child (CBCL) [73] at T0, T4 and T5. Some questions are slightly adjusted to make them more developmentally sensitive for young adults above the age of 18 (e.g., “*I cut classes or skip school*” will be adjusted to “*I cut classes or skip school/work*”). The YSR contains 69 items and the CBCL contains 74 items that are rated on a 3-point Likert scale ranging from 0 = “*not true*” to 2 = “*very true or often true*”. The psychometric properties of the YSR and CBCL are adequate [72, 73].

A short questionnaire measuring psychopathology, which is based on the YSR, the Behavior and Feeling Survey (BFS) [74], will be administered during all six assessments to register changes in psychopathology during the intervention. The BFS contains 12 items that are rated on a 5-point Likert scale ranging from 0 = “*not*” to 4 = “*always*”. The psychometric properties of the BFS are adequate [74].

The Severity Indices of Personality Problems – short form (SIPP-SF) will be used to measure *personality problems* (i.e., degree of self-control, identity integration, responsibility, relational capacities and social concordance) at T0, T2, T4 and T5 (derived from the SIPP–118) [75]. The SIPP-SF contains 60 questions that are rated on a 4-point Likert scale ranging from 0 = “*fully disagree*” to 3 = “*fully agree*”. The psychometric properties of de SIPP-SF are adequate [76].

*Global functioning* will be rated by two informants. Therapists will rate the global functioning of the TAY on the Children Global Assessment Scale (CGAS) [77, 78] at T0, T2, T4 and T5. TAY will rate their own global functioning by means of the Outcome Rating Scale (ORS) [79] at all six assessments. The CGAS is a numeric scale from 1 = “*needs constant supervision*” to 100 = “*superior functioning in all areas*” that is used by mental health care workers to rate the general functioning of youth. The description of the scale has been slightly adjusted to make it more developmentally sensitive for young adults above the age of 18 (e.g., “*no problems at school*” will be adjusted into “*no problems at school/work*”). The CGAS was found to be reliable between raters and across time. Moreover, it demonstrated both discriminant and concurrent validity [78]. The ORS consists of four numeric scales ranging from 1 to 10 in which TAY rate how well they have been doing in four life areas (i.e., individually, interpersonally, socially and overall). The ORS represents a balanced trade-off between the reliability and validity of longer measures [79]. Moreover, the ORS has a moderately high to high internal consistency and can be used as a screening measure and monitoring tool for subjective symptoms of psychological distress [80].

For this study we developed the Individual and Societal Impact Questionnaire (ISIQ) [81] to measure the TAY’s *individual and societal functioning* at T0, T4 and T5. The questionnaire contains 10 questions about the following areas: physical health, mental health, personal hygiene, school and work, friendships, romantic relationships, family, daily activities, independence and leisure time. For each area the following question will be asked: “*Within the last two weeks, how much difficulty did you have with..”.* All questions are answered on a 6-point Likert scale ranging from 0 = “*none*” to 5 = “*a lot*”.

To measure *quality of life* as expressed in quality adjusted life years (QALYs), the Dutch version of the EuroQol 5 Dimension Questionnaire (EQ-5D adolescent and parent version) [82] will be administered at T0, T2 (only for TAY), T4 and T5. The EQ-5D assesses five dimensions: mobility, self-care, usual activities, pain/discomfort and anxiety/depression. Participants are asked to indicate whether they/their children experience “*none”*, “*a little”*, or “*a lot” *of problems in the aforementioned domains. Furthermore, participants are asked to rate their/their child’s health on a scale ranging from 1 = “*the worst imaginable health state*” to 100 = “*the best imaginable health state*”. The psychometric properties of both the TAY and parent version have been established in earlier research [83, 84].

*Stress* will be measured using the Perceived Stress Scale (PSS-10) [85, 86] at T0, T2, T4 and T5. The PSS contains 10 questions that are rated on a 5-point Likert scale ranging from 0 = “*never*” to 4 = “*very often*”. The psychometric properties of the PSS are acceptable [87]. At T1 and T3 a short version of the PSS will be used, the PSS-4 [85]. Also the PSS-4 was found to have acceptable psychometric properties [85, 88]. Based on factor analysis we added one extra question (i.e., the question with the highest factor loading) to the PSS-4.

*Treatment Satisfaction* will be assessed with the Service Satisfaction Scale (SSS) [89] at T5. The SSS contains 4 items that are rated on a 4-point Likert scale ranging from 0 = “*disagree*” to 3 = “*agree*”. The psychometric properties of the SSS are considered as good [89].

Information about *drop-outs* will be gathered with a short drop-out questionnaire containing 5 questions (i.e., reason for drop-out, date of drop out, number of sessions followed, person who initiated to stop the treatment) reported by the therapist.

The quality of the *therapeutic alliance* will be measured with the Therapy Alliance Scale for Adolescents (TASC) [90] at T1, T2, T3 and T4. Both the TAY and the therapist are used as informants. The TASC contains 12 questions that are rated on a 4-point Likert scale ranging from 0 = “*this doesn’t suit me*” to 3 = “*this suits me very well*”. The internal consistency of the TASC has been qualified as good [91].

#### Moderators

*Demographic information* of the TAY, parents/caregivers and therapists will be gathered at T0, T4 and T5 by asking questions about gender, age, ethnicity, education level, work experience and income.

*Type and severity of problems* will also be measured with the SCID-5 Junior [71], YSR [72] and the CBCL [73] at T0, T4 and T5.

*Psychopathology of parents/caregivers* will be measured with the Adult Self-Report (ASR) [92] at T0, T4 and T5. The ASR contains 69 items that are rated on a 3-point Likert scale ranging from 0 = “*not true*” to 2 = “*very true or often true*”. The psychometric properties of the ASR are considered as good [92].

At T0, *treatment expectancy* will be measured with the Parent Expectancies for Therapy Scale (PETS) [93], which is revised for TAY. The PETS contains 7 items that are rated on a 6-point Likert scale ranging from 0 = “*fully disagree*” to 5 = “*fully agree*”. This questionnaire has also been used in previous research [94].

Information about *previous treatments* (including complementary and self-help treatments) will be gathered with the inventory of History of Treatments (VEHI) [95] at T0. The VEHI contains 8 questions in which participants indicate what previous treatment they received. This questionnaire has also been used in previous research [94].

#### Mediators

*Psychological flexibility* will also be included as a mediator for some of our outcomes (e.g., number of DSM-5 diagnoses). We have chosen to also include psychological flexibility as a mediator, as based on earlier studies, we expect that due to ACT your way, TAY will become more psychologically flexible, which in turn will lead to less psychological problems [52].

*Emotion regulation* will be measured with the Emotion Regulation Skills Questionnaire – Junior (ERSQ) [96] and the FEEL-KJ [97, 98] at T0, T2, T4 and T5. The ERSQ measures a broad set of emotion regulation strategies, including emotional awareness, integration of physical sensations and emotions, emotional clarity, understanding of emotions, influencing emotions, accepting emotions, tolerating emotions, willingness to endure difficult situations to achieve goals and self-support. The ERSQ contains 27 items that are rated on a 5-point Likert scale ranging from 0 = “*not at all*” to 4 = “*(almost) always*”. Research investigating the psychometric properties of the ERSQ is still ongoing, but preliminary results show that the internal consistency and construct validity are good (Vervoort, personal communication, June 2021). At T1 and T3 a short version of the ERSQ will be used. The short version has been developed using factor and reliability analyses. From each subscale, the item with the highest factor loading and item-total correlation were selected (i.e., 9 items in total).

The FEEL-KJ measures a broad set of emotion regulation strategies that are used in response to anger, sadness and anxiety. The FEEL-KJ contains 90 questions (30 questions for each emotion) that are rated on a 5-point Likert scale ranging from 0 = “*almost never*” to 4 = “*almost always*”. In the current study, we will ask participants to answer the 30 questions solely for their most extreme emotion (i.e., either anger, sadness or anxiety). The FEEL-KJ has adequate psychometric properties [99].

*Self-compassion* will be measured with the Self-Compassion Scale Short Form (SCS-SF) [100–102] at T0, T2, T4 and T5. The questionnaire contains 12 items that are answered on a 7-point Likert scale ranging from 0 = “*seldom or never*” to 7 = “*almost always*”. The psychometric properties of the SCS-SF have been established in earlier research [100, 101].

The Autonomy Adolescent Questionnaire (AAQ) [103] will be used to measure *autonomy* at T0, T2, T4 and T5. The AAQ divides autonomy into three main categories: attitudinal, emotional and functional autonomy. The AAQ contains 18 items that are rated on a 5-point Likert scale ranging from 0 = “*not all true for me*” to 4 = “*totally true for me*”. The factor structure, convergent validity and divergent validity of the AAQ has been supported in earlier research [103].

*Perfectionism* will be measured with the Frost Multidimensional Perfectionism Scale (FMPS) [104] at T0, T2, T4 and T5. The FMPS consists of 35 questions that are rated on a 5-point Likert scale ranging from 0 = “*strongly disagree*” to 4 = “*strongly agree*”. The reliability and validity of the FMPS is established in earlier studies [104]. In the current study, we only use the subscales: personal standards, organization, concern over mistakes and doubt about actions, containing 26 questions in total. To reduce the burden for TAY, we excluded the subscales: parent expectations and parental criticism as we believe that how TAY view their parents’ expectations and criticism will not be targeted within the intervention.

*Self-esteem* will be measured with the Rosenberg Self-Esteem Scale (RSES) [105, 106] at T0, T2, T4 and T5. The RSES contains 10 questions that are rated on a 4-point Likert scale ranging from 0 = “*totally agree*” to 3 = “*totally disagree*”. Earlier research demonstrated the validity and test- retest reliability of the Dutch RSES [105].

For TAY who receive ACT your way or TAU in a group, *group cohesion* will be measured with the Group Climate Questionnaire-Short (GCQ-S) [107] at T1, T2, T3 and T4. The GCQ-S was revised for TAY. The questionnaire contains three subscales (i.e., Engagement, Conflict and Avoidance) and 12 items that are answered on a 7-point scale ranging from “*totally disagree*” to “*totally agree*”. The reliability and validity of the GCQ-S has been demonstrated as good [108, 109].

#### Other characteristics

*Treatment integrity* will be established by video/audio recording two randomly chosen ACT your way sessions per client (in the case of individual therapy) or group (in the case of group therapy). The sessions will be rated by an independent researcher with a checklist that has been developed in an earlier study [44]. The checklist is based on ACT literature and input from various ACT experts. The checklist can be used to assess which of the six core processes of ACT (i.e., acceptance, defusion, contact with the present moment, self as context, values and committed action) and ACT techniques (i.e., psychoeducation, mindfulness exercises, metaphors and experiential exercises) are carried out per session and if the goal of the session is obtained.

*The content of TAU* will be measured with a short version of the Therapy Procedure Checklist (TPC) [110] at T1, T2, T3 and T4. For each client, therapists fill out how many sessions took place after the previous assessment moment. Then, for each session, therapists indicate which techniques they used in the session (e.g., CBT or psychoeducation), if the session was individual or in a group and if the session was online or offline (for TAU only).

The Life Event Scale (LES) [111] will be used to gather information about several *life events* at T0, T4 and T5. The LES contains 23 questions about various life events (e.g., drug abuse, bereavement, maltreatment and suicide attempts). For each life event, participants are asked to indicate when the event took place (i.e., “*last week”, “last year”, “more than one year ago”*) and to what extent the event made them upset (i.e., ranging from 0 = “*not*” tot 3 = “*very*”). The LES has been used in previous research [94].

*Costs* will be measured with a cost diary based on the Trimbos Institute and Institute of Medical Technology Assessment Questionnaire on Costs Associated with Psychiatric Illness (TiC-P) [112] and PRODISQ [113]. With the cost diary, we will register direct healthcare costs (e.g., visits to healthcare professionals or alternative care), direct non-healthcare costs (e.g., formal and informal care), indirect costs (e.g., productivity losses caused by absence and reduced productivity and school) and out of pocket costs (e.g., transport or parking costs). The cost diary has been used in previous research (e.g., [114]).

### Data analysis

Both intent-to-treat and per-protocol analyses will be executed. Possible baseline differences in demographic characteristics, diagnostic characteristics and psychological flexibility between participants in the ACT your way and TAU condition will be checked by performing t-test (continuous variables) and chi-square analyses (categorical variables). Variables that differ between both conditions will be entered as covariates in all analyses.

#### Effectiveness

The effect of the intervention on the primary (i.e., psychological flexibility and number of DSM-5 diagnoses) and secondary outcomes (i.e., presence of the primary DSM-5 diagnosis, psychopathology, personality problems, global functioning, individual and societal functioning, quality of life, stress, treatment satisfaction, drop-out and therapeutic alliance) will be evaluated using a two-level multilevel analysis in Mplus [115]. Specifically, we will investigate, if condition (i.e., ACT or TAU) predicts the post-intervention (T4) and 6-month follow-up (T5) levels of our primary and secondary outcomes, while controlling for the baseline levels of the concerning outcomes. We will perform a two-level multilevel analysis as we expect that TAY will be nested within therapists. Hence, the first level will represent the TAY and the second level will represent the therapists. We do not expect variance at the measurement level, as the analyses will be performed separately for the post-intervention and 6-month follow-up assessments. Although we expect that therapists will be nested within institutions, we will not include a third level as the number of institutions in our study is too small (i.e., 14 institutions). Instead, in all analyses, we will add dummies representing the mental healthcare institutions.

#### Cost-effectiveness

Cost-effectiveness analysis will be conducted by comparing costs and effects between the ACT your way and TAU condition. The economic evaluation will be executed in accordance with the international guidelines [116]. The cost-effectiveness analyses will be done separately from the perspective of mental health and society over a period of 6 months. The effects of societal costs will be expressed in years to live, corrected for quality of life (QALYs). The costs of ACT you way versus TAU will be expressed in incremental costs per QALY and incremental costs per TAY with the number of diagnoses.

#### Moderation and mediation

To examine if intervention effects are moderated by demographic characteristics (i.e., gender, age, education level and ethnicity), type and severity of problems, psychopathology of parents/caregivers, treatment expectancy and previous treatments, interaction terms will be added to the multilevel model [115].

To investigate if the intervention effects on the primary and secondary outcomes are mediated by psychological flexibility (only for outcomes other than psychological flexibility), emotional regulation, self-compassion, autonomy, perfectionism, self-esteem and group-cohesion at T1, T2 and/or T3, we will perform path-analyses in Mplus [115]. In the analyses we will use a bootstrap procedure and control for the dependency in our data with the “type is complex” and “ML robust estimator” commands.

#### Treatment modality

To explore if there are differences in (cost-)effectiveness between both treatment modalities (i.e., group format and individual format), we will perform some additional analyses. Specially, we will compare TAY who received ACT your way in a group with TAY who received ACT your way individually. Moreover, to rule out treatment modality effects, we will compare TAY who received ACT your way in a group with TAY who received TAU in a group and TAY who received ACT your way individually with TAY who received TAU individually.

## Discussion

The current study protocol describes the design of a multi-center RCT in which we will evaluate the effectiveness and cost-effectiveness of ACT your way compared to TAU. The study will be conducted within a relatively large sample of TAY with diverse psychological disorders. Besides studying the (cost-)effectiveness, we will also investigate for whom and under what circumstances (i.e., moderation analyses) and how (i.e., mediation analyses) ACT your way is (most) effective.

The presented study design has some potential challenges that could turn into limitations if not handled properly. Hence, for each potential challenge, a solution to overcome this challenge will be discussed. First, in the current study, ACT your way will be carried out both in an individual as in a group format, making it more difficult to isolate intervention effects from treatment modality effects. We decided to carry out ACT your way both individually as in groups, as both treatment modalities have their own potential advantages. For instance, group interventions might provide more opportunities for normalization, positive peer modeling and social support, whereas within individual interventions, therapists can more easily adapt exercises and subjects to the specific wishes and needs of each client. To isolate intervention from treatment modality effects, we will perform additional analyses in which we explore possible differences between both treatment modalities (see Method section). Second, the TAU condition might be heterogeneous in content and duration, which makes it challenging to compare the effects of ACT your way with the effects of TAU. To address this challenge, we will ask all therapists in the TAU condition to write down the content and duration of each session. Subsequently, we will investigate if there are differences in duration between ACT your way and TAU. If there are differences, the duration of treatment will be added as covariate in all analyses. Third, recruiting a sample of 140 TAY in 24 months could be challenging. To reach this sample size, we will cooperate with fourteen mental health care institutions in the Netherlands and Belgium. Moreover, to promote participation, we will give TAY a 30 euro giftcard as a reward and only use online questionnaires that TAY, parents/caregivers and therapists can easily fill out at home. Last, non-response and/or drop-out can also be a potential challenge. To minimize non-response, we built a website, including a system that automatically sends research assistants weekly updates via mail, that helps research assistants to monitor the assessment progress. At the website and in the weekly updates, research assistants can easily see which questionnaires are filled out or not and which ones are planned. With the help of this website and automatic mailing system, researchers can directly react to non-response and increase the response rate of the study. To minimize measurement drop-out, we will motivate TAY to continue participating by sending them thank you emails and newsletters and handing out a 5 euro gift card after each assessment. Moreover, when participants drop out of treatment, we will still ask them to fill out the post-intervention and follow-up assessments. Furthermore, to avoid potential bias due to drop-out, all analyses will be carried out according to the intention to treat principle.

Notwithstanding the potential challenges, the study has some important strengths. First, this study will be carried out in a transdiagnostic sample of clinically referred TAY. By recruiting a diverse sample of TAY with various forms of psychopathology (e.g., anxiety disorder, OCD, trauma, depressive disorder, ODD, CD), the results of the study will be generalizable to a large population of clinically referred TAY. Second, the study will be executed within fourteen mental health care institutions in the Netherlands and Belgium. By conducting the study under these real life conditions, the study will have a large ecological validity and will allow us to make conclusions about the real-world (cost-)effectiveness of ACT your way for TAY. Third, in contrast to many earlier studies examining the effects of ACT in youth, we will use a rigorous research design. More specifically, we will conduct an RCT, compare the effects of ACT your way with an active control condition, investigate both short and long term effects of ACT and use a multi-informant (i.e., TAY, parents/caregivers and therapists) and multi-method (i.e.,, questionnaires and diagnostic interviews) approach. When appropriately conducted, RCT’s are seen as the golden standard for intervention research [63] making it is possible to make more grounded conclusions about the (cost-)effectiveness of ACT your way. Moreover, by using an active control condition we are able to say something about the (cost-)effectiveness of ACT compared to routine care. This is more useful than comparing ACT to a waitlist or medication only condition. Furthermore, due to the six month follow-up measure, we can also examine the long term effects of ACT, including possible sleeper effects or recurrence of psychological problems. In addition, using a multi-informant and multi-method approach will decrease the risk for common method bias [117].

Besides the strengths, the study also has some important implications for clinical practice. First, ACT your way is specifically developed for TAY. Developing interventions specifically for TAY and studying the effects of such interventions is clinically important as, compared to other age groups (i.e., younger adolescents and older adults), TAY experience more psychological problems and are less likely to receive age-appropriate treatments. Developing and investigating the effects of interventions for TAY can contribute to improving the continuity of mental health care from adolescence to young adulthood. Second, ACT your way is a transdiagnostic intervention. Examining the effects of transdiagnostic interventions is clinically relevant as they have potential benefits over traditional interventions. Particularly, within transdiagnostic interventions, various psychological symptoms can be treated within one intervention which potentially contributes to the efficiency and (cost-) effectiveness of mental health care. Moreover, transdiagnostic interventions seem specifically relevant for TAY, as comorbid problems, shifting symptom profiles and changing therapy needs are often present during this developmental phase [18, 19]. Third, to our knowledge, this will be the first study investigating the cost-effectiveness of ACT in TAY. Although there are few studies that investigated the cost-effectiveness of ACT in adult samples (e.g., [118]), no cost-effectiveness studies have been conducted in youth. Cost-effectiveness studies are important to inform policy makers about the specific costs and benefits of certain interventions. Last, besides studying the (cost-) effectiveness of ACT, we will also investigate potential moderators and mediators. Studying moderation effects can inform clinical practice for whom and under what circumstances ACT your way is most effective. Moreover, if ACT your way is effective, then it is scientifically and clinically relevant to understand through which mechanisms ACT your way works (mediation analyses). Moreover, by investigating these mechanisms, we can test the theory behind ACT.

## Data Availability

The data obtained in the current study will be available from the last author, Denise Bodden, on reasonable request after publication of the results on the main research questions.

## Abbreviations

ACT: Acceptance and Commitment Therapy
AFQ-Y: Acceptance and Fusion Questionnaire for Youth
ASR: Adult Self-Report
AAQ: Autonomy Adolescent Questionnaire
BFS: Behavior and Feeling Survey
CBCL: Child Behavior Checklist
CGAS: Children Global Assessment Scale
CBT: Cognitive behavioral therapy
CD: Conduct disorder
ERSQ: Emotion Regulation Skills Questionnaire – Junior
EQ-5D: EuroQol 5 Dimension Questionnaire
FMPS: Frost Multidimensional Perfectionism Scale
CCQ-S: Group Climate Questionnaire-Short
ISIQ: Individual and Societal Impact Questionnaire
VEHI: Inventory of History of Treatments
LES: Life Event Scale
OCD: Obsessive–compulsive disorder
ODD: Oppositional defiant disorder
ORS: Outcome Rating Scale
PETS: Parent Expectancies for Therapy Scale
PSS: Perceived Stress Scale
QALY: Quality adjusted life years
RCT: Randomized controlled trial
RSES: Rosenberg Self-Esteem Scale
SCS-SF: Self-Compassion Scale Short Form
SSS: Service Satisfaction Scale
SIPP-SF: Severity Indices of Personality Problems – short form
SCID-5-Junior: Structured Clinical Interview for DSM-5 Disorders—Junior
TASC: Therapy Alliance Scale for Adolescents
TPC: Therapy Procedure Checklist
TAY: Transitional-age youth
TAU: Treatment as usual
YSR: Youth Self Report

## References

[1] Sawyer SM, Afifi RA, Bearinger LH, Blakemore S-J, Dick B, Ezeh AC (2012). Adolescence: a foundation for future health. Lancet.

[2] Ploegmakers-Burg M, Stortelder F (2008). De adolescentie als reorganisatiefase. Tijdschrift voor Psychotherapie.

[3] Kessler RC, Berglund P, Demler O, Jin R, Merikangas KR, Walters EE (2005). Lifetime prevalence and age-of-onset distributions of DSM-IV disorders in the national comorbidity survey replication. Arch Gen Psychiatry.

[4] Dutch Central Bureau for Statistics [Centraal Bureau voor Statistiek]. Mentale gezondheid jongeren afgenomen. [posted 2022 June; cited 2022 Oct]. Available from: https://www.cbs.nl/nl-nl/nieuws/2022/22/mentale-gezondheid-jongeren-afgenomen.

[5] Whiteford HA, Degenhardt L, Rehm J, Baxter AJ, Ferrari AJ, Erskine HE (2013). Global burden of disease attributable to mental and substance use disorders: findings from the global burden of disease study 2010. Lancet.

[6] Costello EJ, Maughan B (2015). Annual research review: optimal outcomes of child and adolescent mental illness. J Child Psychol Psychiatry.

[7] Fergusson DM, Boden JM, Horwood LJ (2007). Recurrence of major depression in adolescence and early adulthood, and later mental health, educational and economic outcomes. Br J Psychiatry.

[8] Woodward LJ, Fergusson DM (2001). Life course outcomes of young people with anxiety disorders in adolescence. J Am Acad Child Adolesc Psychiatry.

[9] Patel V, Flisher AJ, Hetrick S, McGorry P (2007). Mental health of young people: a global public-health challenge. Lancet.

[10] Copeland WE, Shanahan L, Davis M, Burns B, Angold A, Costello EJ (2015). Untreated psychiatric cases increase during the transition to adulthood. Psychiatric Serv.

[11] Olfson M, Marcus SC, Druss B, Pincus HA (2002). National trends in the use of outpatient psychotherapy. Am J Psychiatry.

[12] Edlund MJ, Wang PS, Berglund PA, Katz SJ, Lin E, Kessler RC (2002). Dropping out of mental health treatment: patterns and predictors among epidemiological survey respondents in the United States and Ontario. Am J Psychiatry.

[13] Anderson JK, Newlove-Delgado T, Ford TJ (2022). Annual research review: a systematic review of mental health services for emerging adults - moulding a precipice into a smooth passage. J Child Psychol Psychiatry.

[14] Signorini G, Singh SP, Marsanic VB, Dieleman G, Dodig-Curkovic K, Franic T (2018). The interface between child/adolescent and adult mental health services: results from a european 28-country survey. Eur Child Adolesc Psychiatry.

[15] Davis M, Koroloff N, Ellison ML (2012). Between adolescence and adulthood: rehabilitation research to improve services for youth and young adults. Psychiatr Rehabil J.

[16] Wilens TE, Rosenbaum JF (2013). Transitional aged youth: a new frontier in child and adolescent psychiatry. J Am Acad Child Adolesc Psychiatry.

[17] Delman J, Clark JA, Eisen SV, Parker VA (2015). Facilitators and barriers to the active participation of clients with serious mental illnesses in medication decision making: the perceptions of young adult clients. J Behav Health Serv Res.

[18] Marchette LK, Weisz JR (2017). Practitioner review: empirical evolution of youth psychotherapy toward transdiagnostic approaches. J Child Psychol Psychiatry.

[19] Chu BC (2012). Translating transdiagnostic approaches to children and adolescents. Cogn Behav Pract.

[20] Hayes SC, Luoma JB, Bond FW, Masuda A, Lillis J (2006). Acceptance and Commitment Therapy: model, processes and outcomes. Behav Res Ther.

[21] A-tjak JGL, Davis ML, Morina N, Powers MB, Smits JAJ, Emmelkamp PMG (2015). A meta-analysis of the efficacy of Acceptance and Commitment Therapy for clinically relevant mental and physical health problems. Psychother Psychosom.

[22] Gloster AT, Walder N, Levin ME, Twohig MP, Karekla M (2020). The empirical status of Acceptance and Commitment Therapy: a review of meta-analyses. J Context Behav Sci.

[23] Powers MB, Vörding MBZVS, Emmelkamp PMG (2009). Acceptance and Commitment Therapy: a meta-analytic review. Psychother Psychosom.

[24] Ruiz FJ (2010). A review of Acceptance and Commitment Therapy (ACT) empirical evidence: Correlational, experimental psychopathology, component and outcome studies. Int J Psychol Psychol Therapy.

[25] Substance abuse and mental health services administration. National registry of evidence-based programs and practices [posted 2011; cited 2022]. Available from: https://www.nrepp.samhsa.gov

[26] Fang S, Ding D (2020). A meta-analysis of the efficacy of Acceptance and Commitment Therapy for children. J Context Behav Sci.

[27] Swain J, Hancock K, Dixon A, Bowman J (2015). Acceptance and Commitment Therapy for children: a systematic review of intervention studies. J Context Behav Sci.

[28] Berman MI, Boutelle KN, Crow SJ (2009). A case series investigating Acceptance and Commitment Therapy as a treatment for previously treated, unremitted patients with anorexia nervosa. Eur Eat Disord Rev.

[29] Heffner M, Sperry J, Eifert GH, Detweiler M (2002). Acceptance and Commitment Therapy in the treatment of an adolescent female with anorexia nervosa: a case example. Cogn Behav Pract.

[30] Shabani MJ, Mohsenabadi H, Omidi A, Lee EB, Twohig MP, Ahmadvand A (2019). An iranian study of group Acceptance And Commitment Therapy versus group cognitive behavioral therapy for adolescents with obsessive-compulsive disorder on an optimal dose of selective serotonin reuptake inhibitors. J Obsessive-Compulsive Relat Disorders.

[31] Gauntlett-Gilbert J, Connell H, Clinch J, McCracken LM (2013). Acceptance and values-based treatment of adolescents with chronic pain: outcomes and their relationship to acceptance. J Pediatr Psychol.

[32] Wicksell RK, Melin L, Lekander M, Olsson GL (2009). Evaluating the effectiveness of exposure and acceptance strategies to improve functioning and quality of life in longstanding pediatric pain–a randomized controlled trial. Pain.

[33] Hayes L, Boyd CP, Sewell J (2011). Acceptance and Commitment Therapy for the treatment of adolescent depression: a pilot study in a Psychiatric Outpatient setting. Mindfulness.

[34] Livheim F, Hayes L, Ghaderi A, Magnusdottir T, Högfeldt A, Rowse J (2015). The effectiveness of Acceptance And Commitment Therapy for adolescent mental health: swedish and australian pilot outcomes. J Child Fam stud.

[35] Pahnke J, Lundgren T, Hursti T, Hirvikoski T (2014). Outcomes of an Acceptance And Commitment Therapy-based skills training group for students with high-functioning autism spectrum disorder: a quasi-experimental pilot study. Autism.

[36] Bernal-Manrique KN, García-Martín MB, Ruiz FJ (2020). Effect of Acceptance And Commitment Therapy in improving interpersonal skills in adolescents: a randomized waitlist control trial. J Context Behav Sci.

[37] Azadeh SM, Kazemi-Zahrani H, Besharat MA (2015). Effectiveness of Acceptance and Commitment Therapy on interpersonal problems and psychological flexibility in female high school students with social anxiety disorder. Glob J Health Sci.

[38] Woidneck MR, Morrison KL, Twohig MP (2014). Acceptance and Commitment Therapy for the treatment of posttraumatic stress among adolescents. Behav Modif.

[39] Brown FJ, Hooper S (2009). Acceptance and Commitment Therapy (ACT) with a learning disabled young person experiencing anxious and obsessive thoughts. J Intellect Disabil.

[40] Wang S, Zhou Y, Yu S, Ran L-W, Liu X-P, Chen Y-F (2017). Acceptance And Commitment Therapy and cognitive–behavioral therapy as treatments for academic procrastination: a randomized controlled group session. Res Social Work Pract.

[41] Lee EB, Homan KJ, Morrison KL, Ong CW, Levin ME, Twohig MP (2020). Acceptance and Commitment Therapy for Trichotillomania: a randomized controlled trial of adults and adolescents. Behav Modif.

[42] Masoumian S, Ashouri A, Ghomian S, Keshtkar M, Siahkamary E, Vahed N (2021). Efficacy of Acceptance and Commitment Therapy compared to cognitive behavioral therapy on anger and interpersonal relationships of male students. Iran J Psychiatry.

[43] Livheim F, Tengström A, Andersson G, Dahl J, Björck C, Rosendahl I (2020). A quasi-experimental, multicenter study of Acceptance And Commitment Therapy for antisocial youth in residential care. J Context Behav Sci.

[44] Schraven J, Matthijssen D, Weerden C, Heyne DA, Bodden D (2021). ACT your way: kwaliteit van het protocol en eerste bevindingen van een pilot-effectonderzoek bij adolescenten met een recidiverende depressie. Gedragstherapie.

[45] Stikkelbroek YAJ. Turning depression inside out: life events, cognitive emotion regulation and treatment in adolescents. Utrecht University; 2016.

[46] Arch JJ, Mitchell JL, Genung SR, Judd CM, Andorsky DJ, Bricker JB (2021). Randomized trial of Acceptance And Commitment Therapy for anxious cancer survivors in community clinics: outcomes and moderators. J Consult Clin Psychol.

[47] Cattivelli R, Guerrini Usubini A, Manzoni GM, Vailati Riboni F, Pietrabissa G, Musetti A (2021). ACTonfood. Acceptance And Commitment Therapy-based group treatment compared to cognitive behavioral therapy-based group treatment for weight loss maintenance: an individually randomized group treatment trial. Int J Environ Res Public Health.

[48] Pots W, Trompetter HR, Schreurs KMG, Bohlmeijer ET (2016). How and for whom does web-based Acceptance And Commitment Therapy work? Mediation and moderation analyses of web-based ACT for depressive symptoms. BMC Psychiatry.

[49] Wolitzky-Taylor KB, Arch JJ, Rosenfield D, Craske MG (2012). Moderators and non-specific predictors of treatment outcome for anxiety disorders: a comparison of cognitive behavioral therapy to Acceptance And Commitment Therapy. J Consult Clin Psychol.

[50] Craske MG, Niles AN, Burklund LJ, Wolitzky-Taylor KB, Vilardaga JCP, Arch JJ (2014). Randomized controlled trial of cognitive behavioral therapy and Acceptance And Commitment Therapy for social phobia: outcomes and moderators. J Consult Clin Psychol.

[51] Murillo C, Vo T-T, Vansteelandt S, Harrison LE, Cagnie B, Coppieters I (2022). How do psychologically based interventions for chronic musculoskeletal pain work? A systematic review and meta-analysis of specific moderators and mediators of treatment. Clin Psychol Rev.

[52] Stockton D, Kellett S, Berrios R, Sirois F, Wilkinson N, Miles G (2019). Identifying the underlying mechanisms of Change during Acceptance and Commitment Therapy (ACT): a systematic review of contemporary mediation studies. Behav Cogn Psychother.

[53] Morton J, Snowdon S, Gopold M, Guymer E (2012). Acceptance and Commitment Therapy group treatment for symptoms of borderline personality disorder: a public sector pilot study. Cogn Behav Pract.

[54] Zarling A, Lawrence E, Marchman J (2015). A randomized controlled trial of Acceptance And Commitment Therapy for aggressive behavior. J Consult Clin Psychol.

[55] Ong CW, Barney JL, Barrett TS, Lee EB, Levin ME, Twohig MP (2019). The role of psychological inflexibility and self-compassion in Acceptance And Commitment Therapy for clinical perfectionism. J Context Behav Sci.

[56] Ritzert TR, Berghoff CR, Tifft ED, Forsyth JP (2020). Evaluating ACT processes in relation to outcome in self-help treatment for anxiety-related problems. Behav Modif.

[57] Ong CW, Lee EB, Krafft J, Terry CL, Barrett TS, Levin ME (2019). A randomized controlled trial of Acceptance And Commitment Therapy for clinical perfectionism. J Obsessive-Compulsive Relat Disorders.

[58] Vanzin L, Mauri V, Valli A, Pozzi M, Presti G, Oppo A (2020). Clinical effects of an ACT-group training in children and adolescents with attention-deficit/hyperactivity disorder. J Child Fam stud.

[59] Cartwright J, Hooper N (2017). Evaluating a transdiagnostic acceptance and commitment therapy psychoeducation intervention. Cogn Behav Therapist.

[60] Moradi F, Ghadiri-Anari A, Dehghani A, Reza Vaziri S, Enjezab B (2020). The effectiveness of counseling based on Acceptance And Commitment Therapy on body image and self-esteem in polycystic ovary syndrome: an RCT. Int J Reprod Biomed.

[61] Limburg K, Watson HJ, Hagger MS, Egan SJ (2017). The relationship between perfectionism and psychopathology: a meta-analysis. J Clin Psychol.

[62] Zeigler-Hill V (2011). The connections between self-esteem and psychopathology. J Contemp Psychother.

[63] Schulz KF, Altman DG, Moher D, Group C (2011). CONSORT 2010 statement: updated guidelines for reporting parallel group randomised trials. Int J Surg.

[64] Spybrook J, Bloom H, Congdonc R, Hilld C, Martineze A, Raudenbus S. Optimal design plus empirical evidence. Documentation for the “Optimal Design” Software; 2011.

[65] Baldwin SA, Imel ZE. Therapist effects: findings and methods. In: Lambert MJ, editor. Bergin and Garfield’s handbook of psychotherapy and behavior change. 6th ed. Wiley; 2013. pp. 258–97.

[66] Matthijssen D, de Rooij E, Bodden D. ACT your way: Trainershandleiding: Boom psychologie; 2020.

[67] Matthijssen D, de Rooij E, Bodden D. ACT your way:Boom psychologie; 2018.

[68] Greco LA, Lambert W, Baer RA (2008). Psychological inflexibility in childhood and adolescence: development and evaluation of the avoidance and fusion questionnaire for youth. Psychol Assess.

[69] Simon E, Verboon P (2016). Psychological inflexibility and child anxiety. J Child Fam Stud.

[70] Fergus TA, Valentiner DP, Gillen MJ, Hiraoka R, Twohig MP, Abramowitz JS (2012). Assessing psychological inflexibility: the psychometric properties of the avoidance and fusion questionnaire for youth in two adult samples. Psychol Assess.

[71] Wante L, Braet C, Bögels S, Roelofs J. SCID-5 junior: Gestructureerd klinisch interview voor DSM-5 stoornissen bij kinderen en adolescenten. Boom psychologie; 2020.

[72] Verhulst FC, van der Ende J, Koot JM. Youth Self-Report (YSR): Academic medical center Rotterdam/Erasmus university. Sophia children’s hospital, department of child and adolescent psychiatry; 1997.

[73] Verhulst FC, Van der Ende J, Koot HM. Handleiding voor de CBCL/4–18 [Dutch manual for the CBCL/4–18]. Academic medical center Rotterdam/Erasmus university, Sophia children’s hospital, department of child and adolescent psychiatry; 1996.

[74] Weisz JR, Vaughn-Coaxum RA, Evans SC, Thomassin K, Hersh J, Ng MY (2020). Efficient monitoring of treatment response during youth psychotherapy: the behavior and feelings survey. J Clin Child Adolesc Psychol.

[75] Verheul R, Andrea H, Berghout CC, Dolan C, Busschbach JJ, van der Kroft PJ (2008). Severity indices of personality problems (SIPP-118): development, factor structure, reliability, and validity. Psychol Assess.

[76] Rossi G, Debast I, van Alphen SPJ (2017). Measuring personality functioning in older adults: construct validity of the severity indices of personality functioning - short form (SIPP-SF). Aging Ment Health.

[77] Bunte T, Schoemaker K, Matthys W (2011). Children’s Global Assessment Scale (CGAS).

[78] Shaffer D, Gould MS, Brasic J, Ambrosini P, Fisher P, Bird H (1983). A children’s global assessment scale (CGAS). Arch Gen Psychiatry.

[79] Miller SD, Duncan BL, Brown J, Sparks JA, Claud DA (2003). The outcome rating scale: a preliminary study of the reliability, validity, and feasibility of a brief visual analog measure. J brief Therapy.

[80] Harris R, Murphy MG, Rakes S (2019). The psychometric properties of the outcome rating scale used in practice: a narrative review. J Evid Based Soc Work.

[81] Bodden D, Keulen J, Individual and societal impact questionnaire: Utrecht University; 2021.

[82] EuroQol Group (1990). EuroQol-a new facility for the measurement of health-related quality of life. Health Policy.

[83] Brooks R (1996). EuroQol Group: the current state of play. Health Policy.

[84] Stolk EA, Busschbach JJV, Vogels T (2000). Performance of the EuroQol in children with imperforate anus. Qual Life Res.

[85] Cohen S, Kamarck T, Mermelstein R (1983). A global measure of perceived stress. J Health Soc Behav.

[86] van der Ploeg J, van der Ploeg JD. Stress bij kinderen:Bohn Stafleu van Loghum; 2013.

[87] Lee E-H (2012). Review of the psychometric evidence of the perceived stress scale. Asian Nurs Res.

[88] Vallejo MA, Vallejo-Slocker L, Fernandez-Abascal EG, Mananes G (2018). Determining factors for stress perception assessed with the perceived stress scale (PSS-4) in Spanish and other European samples. Front Psychol.

[89] Bickman L, Athay MM, Riemer M, Lambert EW, Kelley SD, Breda C, et al. Manual of the peabody treatment progress battery. Vanderbilt University; 2010.

[90] Shirk SR, Saiz CC (1992). Clinical, empirical, and developmental perspectives on the therapeutic relationship in child psychotherapy. Dev Psychopathol.

[91] Creed TA, Kendall PC (2005). Therapist alliance-building behavior within a cognitive-behavioral treatment for anxiety in youth. J Consult Clin Psychol.

[92] Achenbach TM, Rescorla LA. Manual for the ASEBA adult forms & profiles: for ages 18–59: adult self-report and adult behavior checklist. University of Vermont, research centre for child, youth and family; 2003.

[93] Kazdin AE, Holland L. Parent expectancies for therapy scale: Yale university. Child Conduct Clinic; 1991.

[94] Stikkelbroek Y, Bodden DHM, Deković M, van Baar AL (2013). Effectiveness and cost effectiveness of cognitive behavioral therapy (CBT) in clinically depressed adolescents: individual CBT versus treatment as usual (TAU). BMC Psychiatry.

[95] Bodden D, Stikkelbroek Y (2010). Vragenlijst Eerdere Hulp en Interventies.

[96] Vervoort L, Boelens E, Berking M, Braet C. Emotion regulation skills questionnaire for children and adolescents:Ghent University; n.d.

[97] Beveren M, Braet C, Cracco E, Theuwis L, Grob A, Smolenski C. FEEL-KJ: vragenlijst over emotieregulatie bij kinderen en jongeren. Hogrefe; 2020.

[98] Braet C, Cracco E, Theuwis L, Grob A, Smolenski C. FEEL-KJ: vragenlijst over emotieregulatie bij kinderen en jongeren. Hogrefe; 2013.

[99] Cracco E, Van Durme K, Braet C (2015). Validation of the FEEL-KJ: an instrument to measure emotion regulation strategies in children and adolescents. PLoS ONE.

[100] Neff KD (2003). The development and validation of a scale to measure self-compassion. Self and Identity.

[101] Raes F, Pommier E, Neff KD, Van Gucht D (2011). Construction and factorial validation of a short form of the self-compassion scale. Clin Psychol Psychother.

[102] Neff KD (2016). The self-compassion scale is a valid and theoretically coherent measure of self-compassion. Mindfulness.

[103] Noom MJ, Deković M, Meeus W (2001). Conceptual analysis and measurement of adolescent autonomy. J Youth Adolesc.

[104] Frost RO, Marten P, Lahart C, Rosenblate R (1990). The dimensions of perfectionism. Cogn Therapy Res.

[105] Franck E, De Raedt R, Barbez C, Rosseel Y (2008). Psychometric properties of the Dutch Rosenberg self-esteem scale. Physiol Belgica.

[106] Rosenberg M. Society and the adolescent self-image. Princeton University Press; 1965.

[107] MacKenzie KR. The clinical application of a group climate measure. International universities press; 1983.

[108] Burlingame GM, Fuhriman A, Johnson JE (2001). Cohesion in group psychotherapy.. Psychother: Theor, Res, Pract, Training.

[109] Johnson JE, Pulsipher D, Ferrin SL, Burlingame GM, Davies DR, Gleave R (2006). Measuring group processes: a comparison of the GCQ and CCI. Group Dynamics: Theory Res Pract.

[110] Weersing VR, Weisz JR, Donenberg GR (2002). Development of the therapy procedures checklist: a therapist-report measure of technique use in child and adolescent treatment. J Clin Child Adolesc Psychol.

[111] Bodden D, Stikkelbroek Y. Life events scale: Utrecht University; 2010.

[112] Hakkaart-van Roijen L, van Straten A, Donker M, Tiemens B. Handleiding Trimbos/iMTA questionnaire for costs associated with psychiatric illness (TiC-P). Instituut voor medical technological assessment, Erasmus MC; 2002.

[113] Koopmanschap MA (2005). PRODISQ: a modular questionnaire on productivity and disease for economic evaluation studies. Expert Rev PharmacoEcon Outcomes Res.

[114] Bodden DH, Dirksen CD, Bogels SM (2008). Societal burden of clinically anxious youth referred for treatment: a cost-of-illness study. J Abnorm Child Psychol.

[115] Muthén LK, Muthén BO. Mplus user’s guide. 8th ed. CA: Muthén & Muthén; 2017.

[116] Ramsey SD, Willke RJ, Glick H, Reed SD, Augustovski F, Jonsson B (2015). Cost-effectiveness analysis alongside clinical trials II—an ISPOR good research practices task force report. Value Health.

[117] Jordan PJ, Troth AC (2020). Common method bias in applied settings: the dilemma of researching in organizations. Aust J Manage.

[118] Finnes A, Enebrink P, Sampaio F, Sorjonen K, Dahl J, Ghaderi A (2017). Cost-effectiveness of Acceptance and Commitment Therapy and a workplace intervention for employees on sickness absence due to mental disorders. J Occup Environ Med.

